# In vivo studies of *Scn5a*+/− mice modeling Brugada syndrome demonstrate both conduction and repolarization abnormalities

**DOI:** 10.1016/j.jelectrocard.2010.05.015

**Published:** 2010-09

**Authors:** Claire A. Martin, Yanmin Zhang, Andrew A. Grace, Christopher L.-H. Huang

**Affiliations:** a Physiological Laboratory, University of Cambridge, Cambridge, United Kingdom; b Department of Biochemistry, University of Cambridge, Cambridge, United Kingdom; c Department of Paediatrics, First Affiliated Hospital, Xi'an Jiaotong University, Xi'an, Peoples' Republic of China

## Abstract

**Objectives:**

We investigate the extent to which the electrocardiographic (ECG) properties of intact *Scn5a+/−* mice reproduce the corresponding clinical Brugada syndrome phenotype and use this model to investigate the role of conduction and repolarization abnormalities in the arrhythmogenic mechanism.

**Methods and Results:**

The ECGs were obtained from anesthetized wild-type and *Scn5a+/−* mice, before and after administration of the known pro- and antiarrhythmic agents flecainide and quinidine. The ECG intervals were measured and their dispersions calculated. *Scn5a+/−* hearts showed ventricular arrhythmias, ST elevation, and conduction disorders including increased QT dispersion, accentuated by flecainide. Quinidine did not cause ventricular arrhythmias but exerted variable effects on ST segments and worsened conduction abnormalities.

**Conclusions:**

The ECG features in an *Scn5a+/−* mouse establish it as a suitable model for Brugada syndrome and demonstrate abnormal conduction and repolarization phenomena. Altered QT dispersion, taken to indicate increased transmural repolarization gradients, may be useful in clinical risk stratification.

## Introduction

The Brugada syndrome (BrS) is associated with increased incidences of polymorphic ventricular tachycardia (VT), exacerbated by flecainide but reduced by quinidine. Two major hypotheses have been suggested for this arrhythmogenicity. On the one hand, a depolarization defect might slow right ventricular (RV) action potential (AP) conduction,^1^ leading to a predisposition to reentrant arrhythmias. Alternatively, alterations in the time course of AP repolarization might result in a shortening of epicardial relative to endocardial AP durations (APDs), thereby increasing repolarization heterogeneities.^2^

These cellular mechanisms are potentially reflected in the electrocardiographic (ECG) features of BrS, of a persistent or transient ST elevation in the right precordial V_1_-V_3_ leads, often in combination with a pattern suggestive of right bundle branch block. This ECG pattern is central to the diagnosis of BrS. Flecainide and other class 1C drugs are often used to unmask the ST elevation,^3^ whereas there are early indications that quinidine can normalize the ST elevation seen in BrS.^4^

A significant proportion of BrS cases are associated with an inherited loss of Na^+^ channel function. This has prompted its recent experimental modeling using a genetic *Scn5a+/−* murine mutant, which shows a 50% reduction in sodium current in cellular studies and higher incidences of arrhythmogenesis compared to wild type (WT) when studied in Langendorff preparations.^5^ Recent studies have demonstrated both a conduction delay and increased repolarization gradients across the RV wall in the *Scn5a+/−* model.^6^ However, thus far, there have been few in vivo ECG studies in murine models whether for *Scn5a+/−* or in other primary electrophysiological disease. Yet, the ECG provides important measures of electrophysiological function, complementing studies of membrane currents at the cellular level and of AP waveforms in whole heart preparations. It has been the gold standard for determining effects of pharmaceutical compounds on cardiac electrophysiology. Furthermore, ECG recordings permit electrophysiological studies in whole animals with intact autonomic inputs to the heart.

Most available ECG studies have used the dog as a predictive preclinical species representing human electrophysiology, but BrS models are limited to pharmacological perfused wedge preparations. The recent extensive introduction of whole animal genetic murine models in studies of arrhythmic disease now requires the extension of ECG techniques to the mouse. Despite differences between murine and human ECG characteristics, particularly in QRS and T-wave morphology, ECG analysis is currently the only noninvasive method of studying cardiac electrophysiology in the intact mouse. Parameters such as the PR and QRS durations can give indications of conduction velocities within the heart, which may also provide indications of tendency toward reentrant arrhythmias. In addition, measurements such as the QT interval, representing the sum of ventricular depolarization and repolarization,^7^ may be used as a noninvasive surrogate for the APD and thereby help clarify the arrhythmogenic mechanism and aid clinical risk stratification.

The present experiments explore the extent to which ECG features in the genetic *Scn5a+/−* murine model reproduce the corresponding clinical phenotype, in particular in its arrhythmic features and characteristic ST elevation. We then use the murine model to explore for alterations in conduction velocity and repolarization times that might thereby be attributed to the *Scn5a+/−* mutation and compare these with features that occur in human BrS. A characterization of these may be useful in future studies assessing the effects of possible pharmacological interventions for BrS, for which the current mainstay of treatment is implanted cardioverter/defibrillator implantation.

## Materials and methods

Mice aged 3 to 6 months were obtained from breeding pairs of heterozygote *Scn5a+/−* and WT inbred 129/sv mice, initially supplied by Harlan (UK). All procedures conformed to the UK Animals (Scientific Procedures) Act 1986. Mice were anesthetized intraperitoneally (IP) using Tribromoethanol (Avertin) (Sigma-Aldrich, Poole, UK) and custom-made chest and limb ECG leads attached. The anesthetic was chosen as it affects hemodynamics and electrophysiology to a lesser extent than other agents such as ketamine.^8^ A baseline ECG recording was obtained, and then either flecainide (20 mg/kg; Sigma-Aldrich) or quinidine (100 mg/kg; Sigma-Aldrich) was injected IP, and an ECG recording made for a 30-minute period. This flecainide dose has been used previously^9^ on both WT mice and mice with an SCN5A-1795insD mutation, in which it resulted in conduction abnormalities, whereas the quinidine dose compares with previous studies using 300 mg/kg IP in electrophysiological studies^10^ or 100 to 150 mg/kg in noncardiological studies.

The ECG signals were amplified and bandpass filtered between 15 and 1000 Hz using Neurolog amplifiers (Preamplifier: Model NL-100; AC amplifier: Model NL104) and filters (Model NL125/126, Digitimer, Welwyn Garden City, Herts, UK), and then digitized using a 1401plus interface (Cambridge Electronic Design, Cambridge, UK). Analysis of ECG waveforms was performed using Spike2 software (Cambridge Electronic Design).

**Fig. 1:**
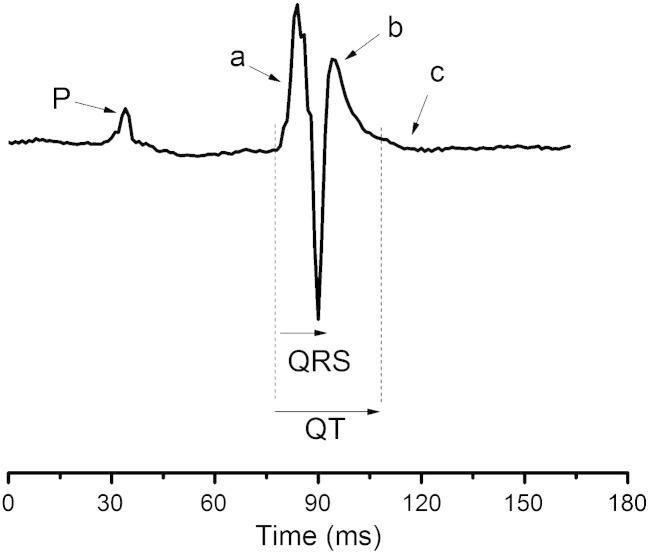
Typical ECG complex from a WT heart before pharmacological manipulation. The murine ECG differs from the human ECG in that the major deflection (a) representing the QRS is followed by a secondary slower deflection (b), and sometimes, a subtle third positive or negative wave (c) follows. We did not consistently find a (c) wave on our ECGs and therefore used the (b) wave to measure the QT interval.

RR, PR, QRS, and QT intervals were measured from the ECG traces. The murine ECG differs from the human ECG in that the major deflection (*a*) representing the QRS is often followed by a secondary slower deflection (*b*) and sometimes a subtle third positive or negative wave (*c*), shown in Fig. 1. Some investigators designate *b* as the T wave and others *c*.^11^ We did not consistently find a *c* wave on our ECGs and therefore used the *b* wave to measure the QT interval, as shown. A heart rate correction factor was used, derived from Mitchell et al^8^ of QTc = QT/(RR/150)^1/2^, as the average RR interval in our mice was 150 milliseconds. Ten measurements of each ECG interval in both chest and limb leads were made for each mouse. Differences in time intervals between hearts were analyzed using Student *t* tests with a modified Bonferroni correction factor.^12^

## Results

Ten WT and 10 *Scn5a+/−* hearts were studied. In each group, 5 were subsequently exposed to flecainide and 5 to quinidine.

### Incidence of ventricular arrhythmia

**Fig. 2:**
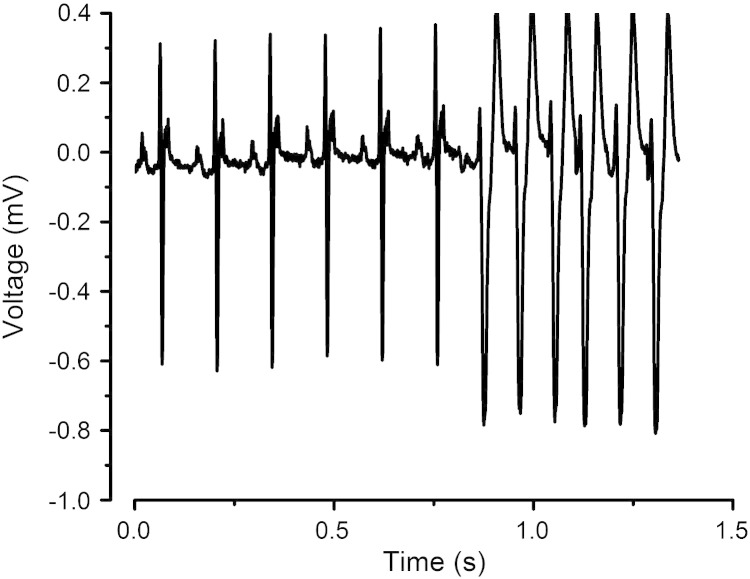
Trace from a typical *Scn5a+/−* heart showing the onset of wide complex tachycardia following the addition of flecainide.

None of the WT hearts studied showed incidences of VT, whether before or after introduction of either flecainide or quinidine. None of the *Scn5a+/−* hearts showed incidences of VT before pharmacological intervention, although 1 *Scn5a+/−* heart demonstrated a bigeminal ECG pattern. However, the addition of flecainide then provoked VT in 2 *Scn5a*+/*−* hearts and nonsustained VT in a further heart (Fig. 2). Addition of quinidine did not lead to any ventricular arrhythmias.

### Presence of ST elevation

**Fig. 3:**
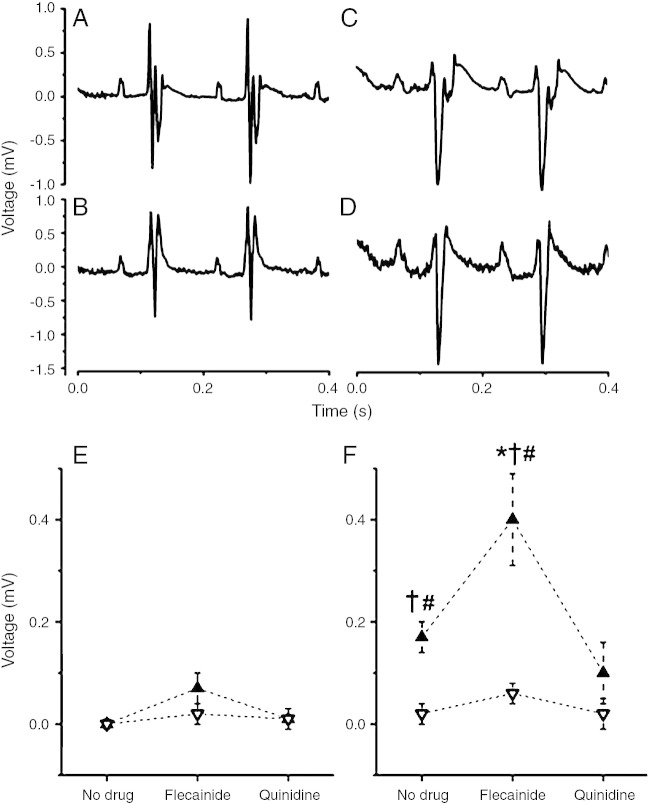
A-D, Traces from chest leads (A, C) and limb leads (B, D) of a *Scn5a+/−* heart showing minor ST elevation before flecainide (A) and accentuated ST elevation after flecainide (C) in the chest leads but no appreciable ST elevation in the limb leads either before (B) or after (D) addition of flecainide. E and F, ST elevation measured from chest and limb leads for WT (E) and *Scn5a+/−* (F) hearts before and after the addition of flecainide or quinidine. Ten WT and ten *Scn5a+/−* hearts were used, with 5 of each exposed to each drug. Results from chest leads are denoted by solid symbols; results from limb leads are denoted by open symbols. ^†^Results of *t* tests comparing WT and *Scn5a+/−* hearts giving values indicating significance. ^⁎^Results of *t* tests comparing values before and after drug in the same hearts giving values indicating significance. ^#^Results of *t* tests comparing values between chest and limb leads in the same hearts giving values indicating significance.

In WT hearts, there was no evidence of ECG ST elevation before pharmacological treatment. However, the addition of flecainide did cause some minor ST-segment elevation up to 0.09 ± 0.03 mV in the chest lead but not in the limb lead. Addition of quinidine did not affect the ST segment. *Scn5a+/−* hearts showed a degree of ST elevation in the chest lead, with a mean of 0.17 ± 0.03 mV. With the addition of flecainide, this significantly increased to a mean ST elevation of 0.40 ± 0.07 mV (Fig. 3). The ST elevation seen in untreated *Scn5a+/−* hearts was reduced to baseline by the addition of quinidine in 2 hearts but actually was slightly increased in 1 heart, from 0.16 to 0.22 mV.

We thus demonstrate the occurrence of ventricular arrhythmias and chest lead ST elevation, particularly after exposure to a flecainide challenge, for the first time in intact anesthetized genetically modified animals used to model BrS. This reproduces the diagnostic clinical ECG features of the human condition and suggests that the murine *Scn5a+/−* mutant would be an appropriate model to explore for and identify those electrophysiological features that could be attributable to a genetic loss of Na^+^ channel function.

### Presence of atrioventricular block

**Fig. 4:**
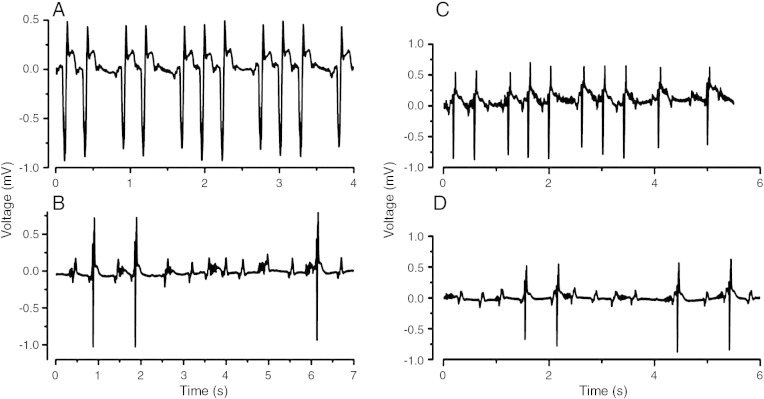
Traces from *Scn5a+/−* hearts after flecainide challenge showing missed beats on a background of ST elevation (A) and high level AV block (B), and after introduction of quinidine showing missed beats (C) and high-level AV block (D).

Untreated WT hearts showed no atrioventricular (AV) block. However, subsequent addition of flecainide and quinidine each caused 1 of 5 hearts to develop 2:1 heart block. Of 5 *Scn5a+/−* hearts, 2 showed short episodes of 2:1 AV block before exposure to flecainide. All 5 *Scn5a+/−* hearts exposed to flecainide showed various degrees of heart block, ranging from missed beats to persistent second-degree block, with 2:1 to 6:1 block, complete heart block, and sinus arrest (Fig. 4A, B). Interestingly, quinidine also produced heart block, with 1 heart showing 2:1 and 3:1 block, and another 3 showing block up to 5:1 (Fig. 4C, D).

### ECG intervals

**Fig. 5:**
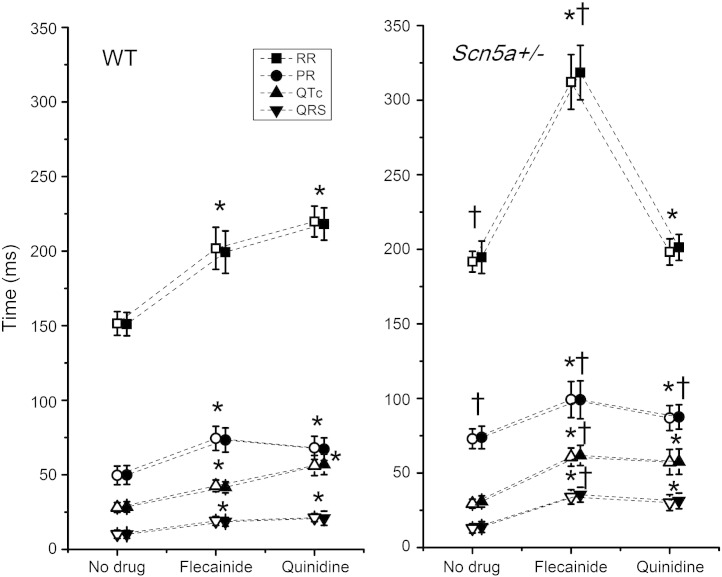
ECG parameters from chest and limb leads measured as RR, PR, QRS, and QTc intervals for WT and *Scn5a+/−* hearts before and after the addition of flecainide or quinidine. Ten WT and ten *Scn5a+/−* hearts were used, with 5 of each exposed to each drug. Results from chest leads are denoted by solid symbols; results from limb leads are denoted by open symbols. ^†^*T* tests comparing WT and *Scn5a+/−* hearts giving values indicating significance. *T tests comparing values before and after drug in the same hearts giving values indicating significance.

Untreated *Scn5a+/−* hearts showed increased RR and PR intervals but statistically indistinguishable QRS and QT intervals compared to WT. Both WT and *Scn5a+/−* mice showed widespread alterations in ECG intervals following the addition of either flecainide or quinidine. Both agents increased ECG RR, PR, QR, and QT intervals (Fig. 5), whether measured in the chest leads or the limb leads. However, flecainide exerted larger effects on these ECG intervals in *Scn5a+/−* than in WT. Consequently, when *Scn5a+/−* and WT were compared a second time in the presence of flecainide, the differences between the two reached significance for all intervals recorded. In contrast, quinidine increased the ECG intervals but did so to a similar degree in both the WT and *Scn5a+/−* hearts, and so the only difference between the two was then in the PR interval. All these effects on cardiac conduction were observed in both the chest lead and the limb lead of the ECG.

### ECG dispersion

**Fig. 6:**
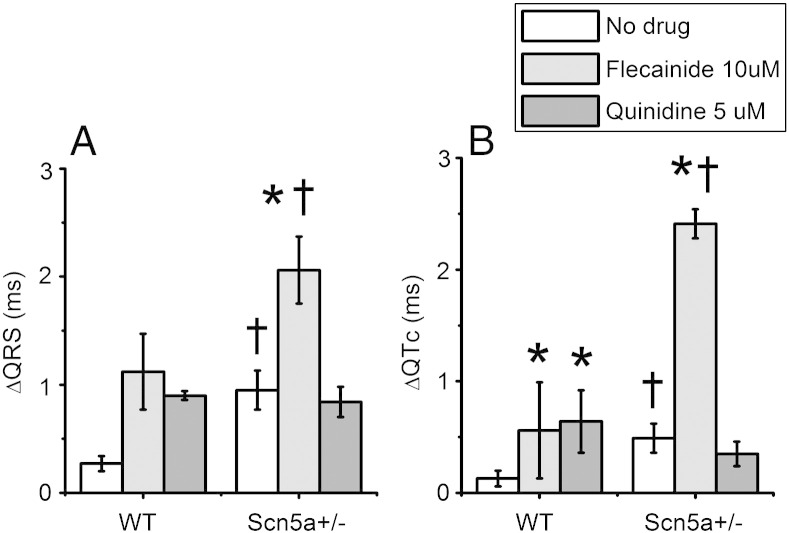
QRS (A) and QTc (B) dispersion measured as the difference in time course in QRS or QTc intervals between chest leads and limb leads. Data are shown for WT and *Scn5a+/−* hearts before and after the addition of flecainide or quinidine. Ten WT and ten *Scn5a+/−* hearts were used, with 5 of each exposed to each drug. ^†^*T* tests comparing WT and *Scn5a+/−* hearts giving values indicating significance. *T tests comparing values before and after drug in the same hearts with values indicating significance.

*Scn5a+/−* traces demonstrated increased dispersions in ECG intervals, attributable to Na^+^ channel loss. Dispersions of ECG intervals within each heart were explored by taking measurements from 2 ECG leads simultaneously—both from a chest lead and a limb lead (analogous to lead II), and the difference between the two in each heart calculated (Fig. 6).

There was an increased difference between limb lead and chest lead in QRS and QTc intervals for *Scn5a+/−* hearts compared to WT hearts. Comparisons of the ECG dispersion in a given heart indicated that in WT hearts both flecainide and quinidine increased the dispersion to a significant level for the QTc intervals. In the *Scn5a+/−* hearts, flecainide increased the dispersion in both the QRS and QTc intervals. In contrast, quinidine did not increase the dispersion in either. Comparing WT and *Scn5a+/−* hearts after the addition of flecainide revealed a further difference in the QRS and QTc dispersions. However, the addition of quinidine served to reduce the difference between WT and *Scn5a+/−* hearts to statistically indistinguishable levels.

## Discussion

BrS is characterized by increased incidences of ventricular arrhythmias exacerbated by flecainide but reduced by quinidine, and with an association of a significant proportion of cases with loss of function mutations involving the Na^+^ channel. The present experiments assessed the extent to which intact anesthetized heterozygotic *Scn5a+/−* mice replicate the ECG features of corresponding in vivo findings to allow them to be used as an experimental model for this condition. We then use this in vivo system to clarify ECG evidence for abnormalities of conduction and repolarization that are specifically attributable to loss of Na^+^ channel function.

### The Scn5a+/− mouse replicates the ECG features of BrS

These ECG studies demonstrated wide complex tachycardias in the *Scn5a+/−* mice, which confirmed previous studies describing VTs in Langendorff perfusion experiments.^6^ They then went on to demonstrate their accentuation by flecainide. This directly parallels its known arrhythmic effects in human VT. In contrast, quinidine exerted an antiarrhythmic effect in direct parallel with both its prevention of phase 2 reentry and of polymorphic VT in experimental canine pharmacological perfused wedge preparations^2^ and its therapeutic antiarrhythmic effects in human BrS.^4^

We then demonstrated for the first time that *Scn5a+/−* mice show an ST elevation specific to the chest lead, which is accentuated by flecainide. This directly correlates with the clinically observed ST elevation and its provocation by flecainide. Both these features are central to the ECG diagnosis of human BrS. The murine *Scn5a+/−* system thus provides an alternative to canine perfused wedge preparations in which ST elevation could only be replicated using multiple drug combinations rather than flecainide alone.^13^ Furthermore, the minor ST elevation in WT hearts with flecainide also replicates clinical reports of Brugada-type ECGs in normal patients treated with flecainide.^14^ Finally, the effect of quinidine on ST elevation is mirrored in studies either showing that quinidine can reverse the ST elevation^4^ or have a variable effect upon the ST interval^15^ in BrS.

The differences in morphology between the human and murine ECGs mean that the ST segment is not identical between the 2 species, and therefore, interpretations about ST-segment elevation must be made with care. It is possible that the ST elevation is a normal variant in the mice; however, the lack of ST elevation in any of the WT mice before drug administration would argue against this. The traces in the BrS mice with flecainide do appear to reproduce the coved appearance of a human BrS type 1 ECG. Furthermore, they resemble the ST elevation seen in murine ECGs after ischemia produced by occlusion of the left anterior descending artery.^16^

### The Scn5a+/− mouse shows ECG evidence for conduction abnormalities directly attributable to loss of Na^+^ channel function

BrS has been associated not only with abnormalities in SCN5A but also mutations in several other genes. Our experiments explored for ECG properties in *Scn5a*+/*−* mice that might identify those features of BrS that might be identified with loss of Na^+^ channel function. These revealed abnormalities in conduction and repolarization.

The intact anesthetized *Scn5a+/−* mice showed features of second-degree AV block and increased PR intervals, consistent with the longer conduction latencies previously observed in Langendorff perfused *Scn5a+/−* hearts.^6^ These findings are directly translatable to clinical observations. Genetically heterogeneous groups of patients with BrS show varying findings of either similar^17^ or longer^18^ PR intervals and AV block; however, BrS patients with an identifiable *SCN5A* mutation have been reported generally to show longer PR intervals and more bradyarrhythmias^19^ than those without an identifiable *SCN5A* mutation. Furthermore, loss-of-function *SCN5A* mutations are associated with a heterogeneous range of conditions including Progressive Cardiac Conduction Disease and Sick Sinus Syndrome and not only with BrS. Together, these findings suggest that ECG features of conduction delay seen in BrS may be attributable specifically to the Na^+^ channel loss of function.

### The conduction abnormalities in Scn5a+/− mouse are exacerbated by flecainide

Flecainide exacerbated the conduction delay in all mice, but particularly in the *Scn5a+/−*. This is consistent with its actions in pharmacological Na^+^ channel block when this is superimposed upon a preexisting reduction in Na^+^ channel function caused by *Scn5a+/−*. These findings directly correlate with clinical studies reporting that flecainide increases PR intervals and QRS durations both in control and BrS patients,^17,20^ with greater effects in the latter.

That quinidine also increased conduction abnormalities might suggest that theoretically it could worsen conduction in cases of BrS with an already diseased conduction system. However, in clinical trials, this has not been seen,^21^ and the effects shown here may be due to the relatively high doses of quinidine, in comparison to the low doses used clinically, where the effects of blocking K^+^ channels are likely to be much stronger that those blocking Na^+^ channels.

### QT interval is not a direct correlate of APD

The clinical QT interval has been taken to be the sum of depolarization and repolarization time courses. The corresponding clinical findings vary, either reporting QTc intervals in patients with BrS within a reference range^22^ or that were significantly longer than controls.^20^ Several studies^17,20^ have shown that the QTc interval is prolonged with the introduction of flecainide both in BrS and control groups, but more so in patients with BrS.

The reduced Na^+^ current in BrS has been suggested to exaggerate the normally existing electrical heterogeneity within the ventricular wall and in particular accentuate the AP notch in the RV epicardium. Pitzalis et al^20^ have suggested that the greater the baseline AP notch, the longer the induced AP prolongation, which should be reflected in prolongation of the QT interval. However, other studies have related electrophysiological abnormalities in BrS with shortening of APDs in the RV epicardium.^2,6^ This contrasts with the normal or slightly prolonged QTc interval seen both clinically and in the present experiments.

It is possible that the QTc does not shorten in parallel with the APD in *Scn5a+/−* hearts owing to the selective nature of the AP shortening in the RV epicardium, with other ventricular regions less affected. The ECG represents a transthoracic interpretation of the electrical vector through the whole myocardium, and therefore, regions showing selective electrical changes are likely to be underrepresented. In contrast, increased transmural gradients of repolarization times may manifest as a prolonged QTc, as electrical repolarization would be recorded across a wider time frame. Danik et al^11^ found that the QTc interval was not correlated with the APD in murine studies.

### QT dispersion as an indication of repolarization heterogeneity

Although there is disagreement to the extent that QT dispersion represents repolarization heterogeneities,^23^ there is evidence that it is useful in risk assessment in ischemia, chronic heart failure, and long QT syndrome. There remains controversy over the usefulness of QT dispersion as a marker of risk in BrS, with studies either supporting or refuting its value.^24,25^ Nevertheless, Na^+^ channel–blocking agents do appear to increase temporal QT dispersion in patients with BrS.^20^ We could not find clinical studies investigating the effect of quinidine on ECG dispersions in BrS. Our findings of heightened QT dispersion in *Scn5a+/−* hearts, further increased by flecainide but not by quinidine, directly parallel the respective pro- and antiarrhythmic effects of the 2 drugs experimentally and clinically. Furthermore, recent studies have implicated increased repolarization gradients across the RV wall in the arrhythmogenic mechanism in *Scn5a+/−* mice, which are increased by flecainide but reduced by quinidine.^6^

In conclusion, this article has extended analysis of BrS from limited pharmacological models in cellular or tissue preparations to a genetic *Scn5a+/−* model using the intact mouse. The model reproduces the clinical Brugada phenotype, lending weight to studies using it to investigate arrhythmogenic mechanisms. We attribute conduction abnormalities in BrS to a Na^+^ channel defect. Localized precordial ST elevation may reflect specific RV repolarization abnormalities. Finally, we show that the QT interval derived from the ECG is not a direct correlate of APD in a specific cardiac region but suggest that QT dispersion, as an indicator of increased transmural repolarization gradients, may be used in clinical risk assessment. Future studies measuring QT intervals simultaneously with monophasic action potentials could further investigate this.
